# Elevational Gradients in Bird Diversity in the Eastern Himalaya: An Evaluation of Distribution Patterns and Their Underlying Mechanisms

**DOI:** 10.1371/journal.pone.0029097

**Published:** 2011-12-13

**Authors:** Bhoj Kumar Acharya, Nathan J. Sanders, Lalitha Vijayan, Basundhara Chettri

**Affiliations:** 1 Division of Conservation Ecology, Sàlim Ali Centre for Ornithology and Natural History, Anaikatty, Coimbatore, India; 2 Department of Ecology and Evolutionary Biology, University of Tennessee, Knoxville, Tennessee, United States of America; University of Copenhagen, Denmark

## Abstract

**Background:**

Understanding diversity patterns and the mechanisms underlying those patterns along elevational gradients is critically important for conservation efforts in montane ecosystems, especially those that are biodiversity hotspots. Despite recent advances, consensus on the underlying causes, or even the relative influence of a suite of factors on elevational diversity patterns has remained elusive.

**Methods and Principal Findings:**

We examined patterns of species richness, density and range size distribution of birds, and the suite of biotic and abiotic factors (primary productivity, habitat variables, climatic factors and geometric constraints) that governs diversity along a 4500-m elevational gradient in the Eastern Himalayan region, a biodiversity hotspot within the world's tallest mountains. We used point count methods for sampling birds and quadrats for estimating vegetation at 22 sites along the elevational gradient. We found that species richness increased to approximately 2000 m, then declined. We found no evidence that geometric constraints influenced this pattern, whereas actual evapotranspiration (a surrogate for primary productivity) and various habitat variables (plant species richness, shrub density and basal area of trees) accounted for most of the variation in bird species richness. We also observed that ranges of most bird species were narrow along the elevation gradient. We find little evidence to support Rapoport's rule for the birds of Sikkim region of the Himalaya.

**Conclusions and Significance:**

This study in the Eastern Himalaya indicates that species richness of birds is highest at intermediate elevations along one of the most extensive elevational gradients ever examined. Additionally, primary productivity and factors associated with habitat accounted for most of the variation in avian species richness. The diversity peak at intermediate elevations and the narrow elevational ranges of most species suggest important conservation implications: not only should mid-elevation areas be conserved, but the entire gradient requires equal conservation attention.

## Introduction

Biodiversity varies geographically, and understanding why is one of the fundamental questions in biogeography, macroecology, and conservation ecology. Perhaps the best- studied pattern in species richness is the latitudinal gradient in diversity - the decline (for most taxa) in richness with increasing distance from the equator [1], [2], [3]. Elevational gradients, though perhaps not studied as intensively as the latitudinal gradient, provide equally striking patterns in diversity [4]. The most common patterns seem to be either decreasing richness with increasing elevation or a hump-shaped pattern, in which diversity peaks at mid-elevations [5], [6]. While many studies have documented patterns in diversity along elevational gradients and have attempted to describe the mechanisms underlying those patterns, the consensus on the generality of pattern and processes is still a topic of discussion [4]. Understanding such patterns and their underlying mechanisms is critically important for conservation efforts [7], especially in biodiversity hotspots, montane regions which are likely to be especially threatened by climate change, and regions that have been generally un- or under-explored by biologists.

Patterns in diversity along elevational gradients might vary among taxa, regions, and spatial scales [8]–[11]. Though the hump-shaped pattern is the most commonly reported pattern, its ubiquity might depend on the methods employed, sampling effort, taxa and gradient considered [5], [9], [12]. Moreover, whether the entire gradient is sampled can also influence the apparent pattern [13]. With some exceptions, studies on elevational diversity gradients are restricted to either low, mid or high elevation, in essence covering only a part of the gradient or on a smaller mountain with narrow elevational breadth. Data that span over the entire gradient or data from the highest elevations where life occurs, especially when the gradient itself is extensive, likely provide more opportunities for better understanding patterns of species richness [9], [14], [15], [16]. For instance, the extensive elevational gradient of Himalaya (from 200 m to >8000 m) provides an ideal test bed for a broader understanding of the pattern of diversity with elevation and the underlying causes of the pattern [17]. Our study, for example, covers 4500 m in elevation (300–4700 m) in the hitherto under-explored Eastern Himalayan Mountains. To our knowledge, this is the most extensive elevational gradient for birds ever examined.

The most frequently documented correlates and drivers of elevational patterns of diversity include contemporary climate (temperature, rainfall; [18], [19], [20]), biological processes (mass effects, productivity, habitat heterogeneity, interspecific interactions; [1], [9], [21], [22]), evolutionary and historical processes (niche conservatism, isolation, speciation, endemism, and evolutionary diversification; [23]–[26] and spatial factors (area and the mid-domain effect; [27]–[30]).

One idea that has persisted in the literature is Rapoport's rule, which, as originally formulated, posited that the mean latitudinal range of species is smaller at low latitudes than at high latitudes because species at high latitudes are adapted to a broad spectrum of climatic conditions [31]. A ‘rescue effect’ then would lead to higher species richness at lower latitudes than at higher latitudes if those species at high latitudes ‘spill’ down to lower latitudes. Some empirical support exists for Rapoport's rule, though the idea is still contentious [32]. Stevens [33] extended Rapoport's rule to apply to elevational gradients as well, such that the ranges of species might be greater at high elevations than at low elevations, and the rescue effect would suggest that richness should decline with elevation. And indeed, there is some empirical support for Rapoport's elevational rule [28], [34], [35].

Another relatively controversial idea is that geometric constraints or mid-domain effects (MDE) are important drivers for such a pattern [29], [36]. MDE results from random placement of species ranges within a bounded geographical domain creating a mid-elevation peak of species richness [29], [37]. Though critics argue that the MDE does not provide biological explanations for elevational richness patterns [38] and some MDE patterns might be spurious [39], the MDE at a minimum provides appropriate null models and should be evaluated in combination with biotic, abiotic and historical factors [30].

In this study, we examine the elevational gradient in bird diversity in the Sikkim region of the Eastern Himalaya, home to the tallest mountains in the world. In particular, our aims are to document, describe, and explain the elevational gradient in bird diversity in the Eastern Himalaya. First, we describe the pattern along this extensive gradient (we note that we did not sample the entire gradient due to logistical reasons but this might not influence overall pattern as there are very few plants or birds above the highest elevation we have sampled). Then, we evaluate a suite of biotic and abiotic factors that might be correlated with bird diversity, focusing on geometric constraints, temperature, precipitation, potential evapotranspiration (PET), actual evapotranspiration (AET), plant species richness, tree density, shrub density and basal area of tree. These parameters broadly represent MDE, energy, productivity and habitat diversity. Finally, we assessed the range size distribution pattern of birds along the elevation gradient by examining the elevational range size of each bird species and the applicability of Rapoport's rule.

## Methods

### Study area

The study area is in the Eastern Himalayan Mountains (the state of Sikkim in India; 27° 03' to 28° 07' N and 88° 03' to 88° 57' E). Elevation in this region ranges from c.300 m to above 8000 m. The study sites were located in the Teesta Valley which consists of rough hilly terrain and varies in elevation from 300–5500 m. Both climate (tropical to temperate) and vegetation type (tropical forest to alpine meadows) vary with elevation, within a distance of ∼150 km. The lower and middle valleys (<2000 m) are hot and humid with annual precipitation exceeding 2500 mm, while elevations above 2500 m are relatively drier and colder with substantially less rainfall (<1000 mm). At the high elevation sites, precipitation is in the form of snowfall, and most of the alpine region remains under snow for almost 7–8 months a year.

Six major vegetation zones occur in the study area. These are Tropical semi-deciduous forests (<900 m), Tropical moist and broad-leaved forests (900–1800 m), Temperate broad-leaved forests (1800–2800 m), Temperate coniferous and broad-leaved forests (2800–3800 m), Sub-alpine (3800–4500 m) and Alpine vegetation (>4500 m) [40].

### Bird sampling

**Figure 1:**
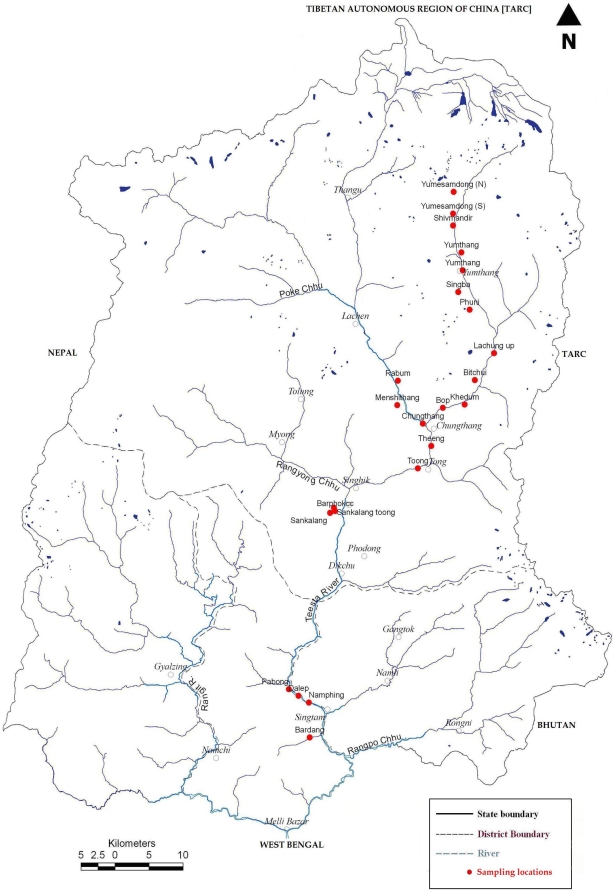
Map of Sikkim showing sampling locations in different elevations.

To quantify variation in the richness and abundance of birds along this elevational gradient, at 22 sites, we used the open width point count method along transects [41]. The open width point count method is particularly effective for rapid assessment of bird assemblages, especially when large areas are sampled [41]. The transects varied in length from 600–1000 m, depending on vegetation type and accessibility, and were distributed among six vegetation types (Figure 1, Table 1). We avoided sites with clear evidence of disturbance by humans. Elevational distance between two consecutive sites was 150 m to 350 m depending upon the accessibility and availability of the sites. Within each transect at each site, we established permanent points (6–10 points depending upon the length of the transect) for bird sampling, keeping a minimum of 100 m distance between two adjacent sampling points along the transect. We conducted a count at each point for five minutes and recorded the identities and abundance of all birds seen or heard. All points were replicated 1–3 times each during winter (Dec-Feb), summer (Mar-May), monsoon (Jun-Aug) and post monsoon (Sept-Nov) during 2003–2006. Thus, a total of 2428 point counts were conducted during entirety of this study. Prior to the field study, we obtained the permission from the Forests, Environment and Wildlife Management Department, Government of Sikkim (Permit Nos. 07/GOS/FEWD and 54/GOS/FEWD).

**Table 1:** Details of transects laid along the elevation gradient of Sikkim, Eastern Himalaya.

Transect	Elevation (m)	Vegetation Types	Latitude(° ′N)	Longitude(° ′E)	Effort
T1	300	TrSDF	27 12.1	88 28.9	14
T2	450	TrSDF	27 14.8	88 27.2	14
T3	600	TrSDF	27 14.3	88 28.4	15
T4	750	TrSDF	27 15.1	88 26.6	14
T5	900	TrSDF	27 29.3	88 30.6	16
T6	1050	TrMBF	27 29.4	88 30.7	16
T7	1200	TrMBF	27 29.5	88 30.2	12
T8	1350	TrMBF	27 33.1	88 38.5	15
T9	1500	TrMBF	27 34.2	88 39.2	15
T10	1650	TrMBF	27 36.2	88 38.6	15
T11	1900	TBF	27 37.6	88 36.9	15
T12	2150	TBF	27 37.7	88 42.2	16
T13	2400	TBF	27 39.8	88 36.3	15
T14	2650	TBF	27 39.5	88 43.7	15
T15	2850	TCF	27 41.1	88 45.4	12
T16	3050	TCF	27 45.2	88 43.8	12
T17	3250	TCF	27 46.8	88 42.5	11
T18	3450	TCF	27 48.4	88 42.7	14
T19	3650	TCF	27 49.3	88 42.5	12
T20	4000	SAV	27 51.4	88 41.6	12
T21	4350	SAV	27 52.2	88 41.7	13
T22	4700	AV	27 54.8	88 41.9	10

TrSDF - Tropical semi-deciduous forests; TrMBF - Tropical moist and broad-leaved forests; TBF - Temperate broad-leaved forests; TCF - Temperate coniferous forests; SAV - Sub Alpine vegetation and AV - Alpine vegetation. Effort - No. of times each transect was repeated for sampling birds.

### Vegetation sampling

We also sampled the trees and shrubs at each of the 22 sites. Along each transect used for sampling birds at each site, we placed 10 20 m × 10 m quadrats for enumeration of trees. Plants with GBH (girth at breast height) >20 cm were considered as trees. For estimating shrub density, two 5 m × 5 m sub-quadrats were placed diagonally within each of the 20 × 10 m quadrats. Thus, for each site, we recorded the richness and density of trees, the richness and density of shrubs, and the GBH of trees. We also estimated basal area of trees for each site using the formula: Basal Area  =  (GBH)^2^/4Л, where Л  = 3.14.

### Climate and climatic variables

We obtained rainfall and temperature data from seven locations at different elevations in the study region from Indian Meteorological Department. Based on these data, rainfall and temperature were estimated for all locations using regression equations, as is often done in these types of analyses [18], [20]. The equations used for estimation were

rainfall =  -0.7909(elevation) + 4046.1, R^2^ = 0.975, p<0.01

temperature  = -0.0062(elevation) + 29.85, R^2^ = 0.983, p<0.01.

We calculated potential evapotranspiration (PET) using the formula [PET =  mean annual bio-temperature (i.e. temperature > 0°C) x 58.93] (see [42]). PET is an estimate of the potential amount of water released through transpiration and surface evaporation from vegetation that is well supplied with water [43] and is considered as a surrogate of energy. We used actual evapotranspiration (AET) as surrogate of productivity. We calculated AET using the Turc's formula, AET = P/ [0.9 + (P/L)^ 2^]^1/2^ with L = 300 + 25T + 0.05T^3^, where P = mean annual precipitation and T = mean annual temperature [18], [44].

### Data Analysis

#### How does richness vary with elevation?

We examined how observed species richness, estimated richness had sampling gone to completion, rarefied richness and density of birds varied with elevation for the 22 sampling sites. Observed species richness was the total count of species detected across all seasons at each site. We followed Reynolds et al. [45] to estimate density as D = n *10000/ Л r^2^C, where D = bird density (numbers/ha), n = total number of birds observed in all counts within the specific radius, r = specific radius (m) (specific radius is the average radial distance of birds from the observer), C = total number of counts conducted and Л = 3.14. We also estimated individual-based rarefied richness, which accounts for variation in the number of individuals sampled. We rarefied to the lowest number of individuals detected in any one survey (n = 260). Because some sites were more frequently sampled than others, we also used sample-based rarefaction (rarefied to lowest number of counts conducted (n = 72 point counts) for any site. Since the individual and sample-based rarefaction results were qualitatively similar, we report only the results from the individual-based rarefaction (but the results from sample-based rarefaction are presented in Figures S1 and S2).

**Figure 2:**
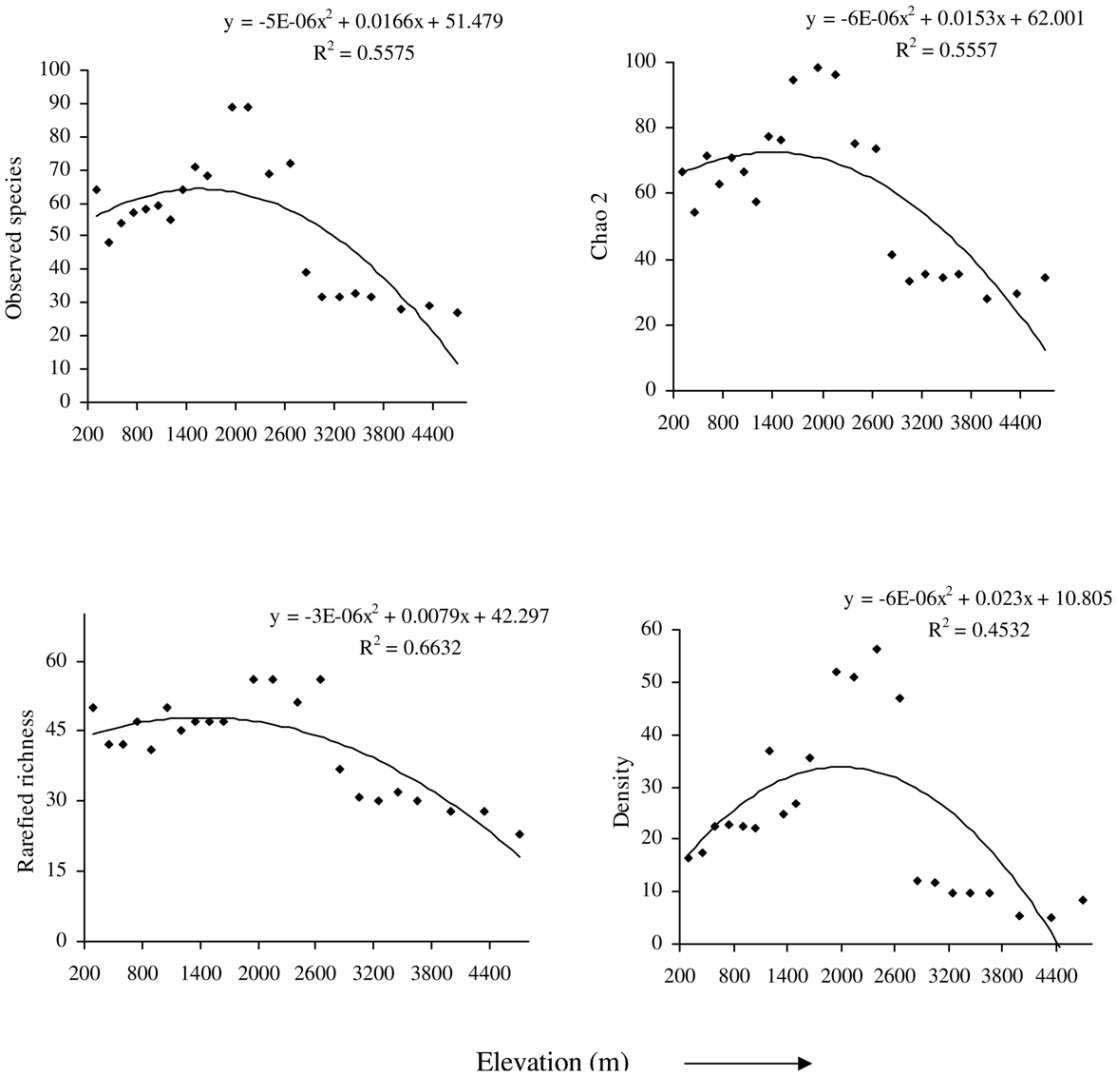
Elevational distribution pattern of birds. Elevational variation of bird species richness (observed and Chao 2), rarefied richness (rarefied to 260 individuals) and density (birds/ha) in Sikkim, Eastern Himalaya.

Additionally, we used two other approaches to assess whether our sampling protocol introduced any potential biases. First, we used a two-step rarefaction approach by using only six sampling points (the lowest number of points sampled) at each site from one season (summer) and rarefied to lowest number of individuals detected at any one site (n = 15). We found that the pattern of this two-step rarefaction procedure did not differ qualitatively from either total bird species richness (see results) or the more standard individual- or sample-based rarefaction procedures described above (see Figure 2 and Figure S3). Second, because the number of species in a sample rarely asymptotes, either because of missed species or because of unequal sampling, we estimated the Chao2 estimated species richness of each site using EstimateS, version 7 [46]. While non-parametric estimators have their own biases and levels of precision, we selected the Chao2 because this estimator is less sensitive to patchiness of species distributions and variability in the probability of encountering species [47].

To describe the pattern of observed species richness, the Chao2 estimate of species richness, rarefied richness and density along the elevational gradient, we used ordinary least squares (OLS) regressions. Because the relationship between any estimate of richness and elevation need not be linear, we also used a quadratic term (elevation^2^) in each model to relate each of the response variables (observed richness, Chao2 estimated species richness, rarefied richness and density) to elevation. We compared AIC values to determine whether the linear or quadratic model best accounted for variation in each of the response variables. Because spatial autocorrelation can inflate errors in the statistical analyses of ecological data [48], [49], we also used spatial regressions. We generated spatial correlograms for observed bird species richness and density using Moran's *I* coefficients with the software SAM version 4.0 (see [50] for application and analytical procedure).

#### Is there evidence of a mid-domain effect?

We used Monte Carlo simulations programme, mid-domain effect null model [30] for testing geometric constraints or mid-domain effects on species ranges. This programme uses empirical range sizes or range midpoints within the elevational range and simulates species richness curves based on analytical-stochastic models [29], [37]. To test the impact of spatial constraints on species richness, 95% prediction curves were produced based on 50,000 simulations (without replacement) using empirical range sizes. Simulations using range mid-points arbitrarily show better fit to null model because midpoint simulations are too constrained by the empirical data [30]. Hence, range size simulation rather than range midpoint simulations are better for assessing fit to MDE null models for geometric constraints of species richness. The empirical species richness curves were compared with the 95% confidence intervals generated from species range sizes. Species richness data were generated at 100 m elevational increments. We then regressed the average of the predicted number of species against the observed empirical values to assess whether geometric constraints could contribute to the pattern of bird species richness in this system. In addition, we also used MDE predicted richness as predictor variable in the multiple regression model (see below).

#### What factors are correlated with richness?

We first used several simple linear regression models to explore the potential of individual environmental factors to predict observed bird species richness, Chao2 estimated species richness, rarefied species richness and density. We then performed stepwise multiple regressions to identify the factors that were related with the species richness and density of birds. Among the set of factors, we selected six variables - AET, MDE predicted richness, plant species richness, tree density, shrub density and basal area of trees. Since temperature, rainfall and PET were highly correlated with one another and with AET, we dropped these factors from the model and used only AET. In each step, the factor with lowest AIC and sums of squares was dropped until we found no significant difference between the model with or without that particular factor. This analysis was performed using statistical package R version 2.11.0. As discussed above, we also generated spatial correlograms for AET, MDE predicted richness, plant species richness, tree density, shrub density and basal area of trees using software SAM version 4.0 (see [50]).

#### Are range size and elevation correlated?

We estimated the range of each species as the difference between the lowest and highest elevation at which that species was recorded during the study. The assumption then is that the species occurs at all intermediate elevations between lowest and highest elevation (see [8], [51]). We then asked whether there was a relationship between range size and elevation by regressing range size of each species against the lower and upper limits of its elevational range, as would be predicted if Rapoport's rule holds in this system.

## Results

### How does richness vary with elevation?

We observed a total of 297 bird species over the course of the study from the 22 sites along this elevational gradient. The number of species observed at a single site varied from 27 to 89. Bird species richness exhibited a mid-elevation peak: the highest number of species was observed at approximately 2000 m (quadratic r^2^ = 0.55, P < 0.01; Figure 2) in the eastern Himalaya.

**Figure 3:**
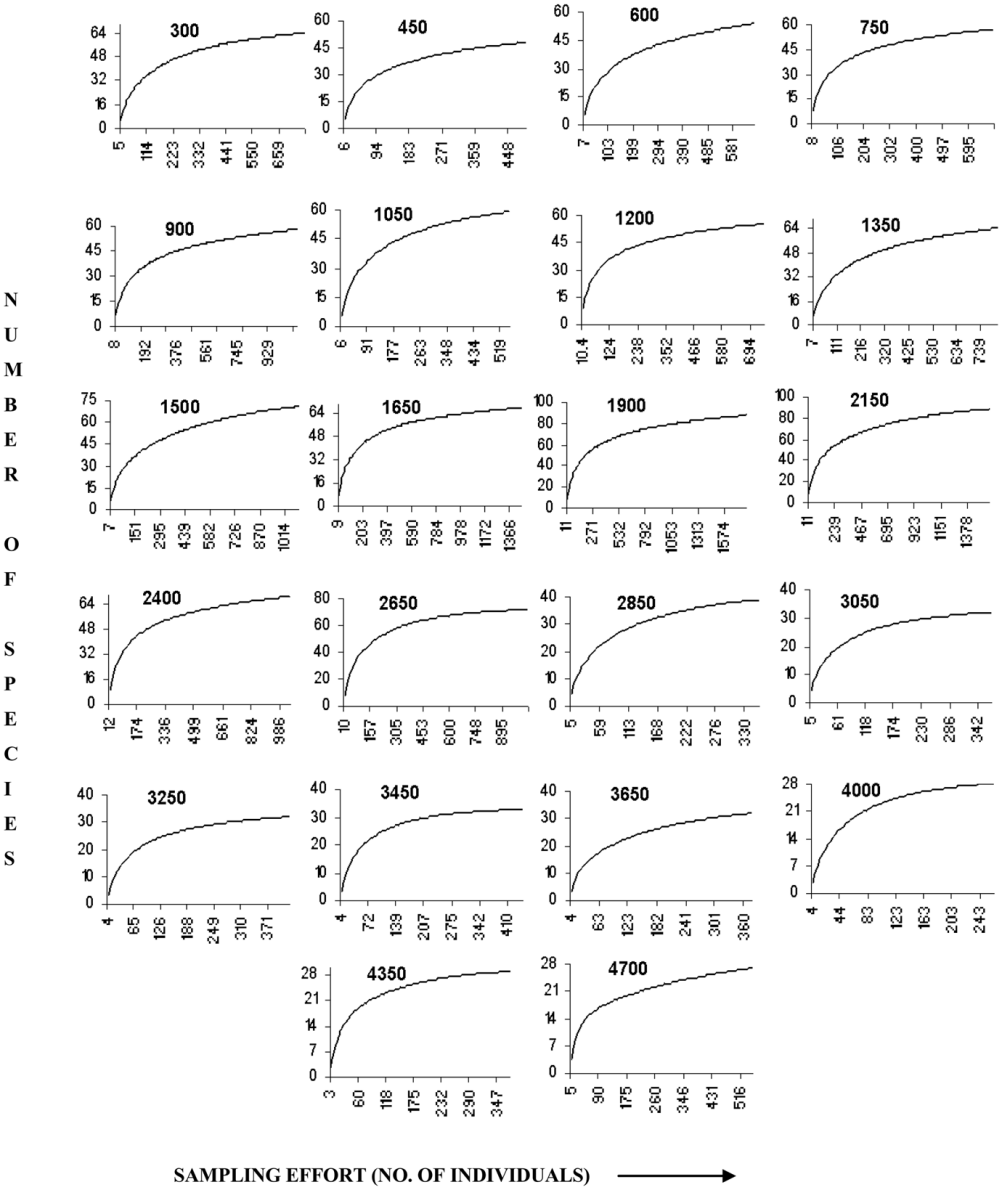
Species accumulation curves of birds. Curves based on number of individuals detected in different elevational transects in Sikkim, Eastern Himalaya. Numbers in the figures indicate elevation (m) of the sampling site.

Although the species accumulation curves approached a plateau for each of the sites, richness did not completely plateau for several of them (Figure 3 and Figure S1). Hence, we also examined the Chao2 estimate of the number of species had sampling gone to completion. Similar to the pattern for observed richness, the Chao2 estimated species richness also peaked at mid-elevations (r^2^ = 0.55; p < 0.01; Figure 2). Because the number of individuals varied among sites, we also examined whether rarefied species richness varied systematically with elevation. The pattern of rarefied richness along the elevational gradient was best explained by a quadratic regression (r^2^ = 0. 66; p < 0.01). However, the pattern was not clearly hump-shaped. Instead, below about 2000 m, there was no systematic variation in rarefied richness with elevation, but above 2000 m, rarefied richness declined with elevation (Figure 2).

The total number of birds encountered varied from 260 to 1964 per site, and the mean number of individuals per point along each transect at each site ranged from 3.31 to 12.87. The density of birds ranged from 5.1 to 56.3 birds ha^-1^ with the maximum density recorded at 2400 m and the minimum at 4350 m. Both the mean number of individuals per point (r^2^ = 0. 46; p < 0.01) and density (r^2^ = 0. 45; p < 0.01) peaked at mid-elevations (Figure 2).

### Vegetation along elevation gradient

**Figure 4:**
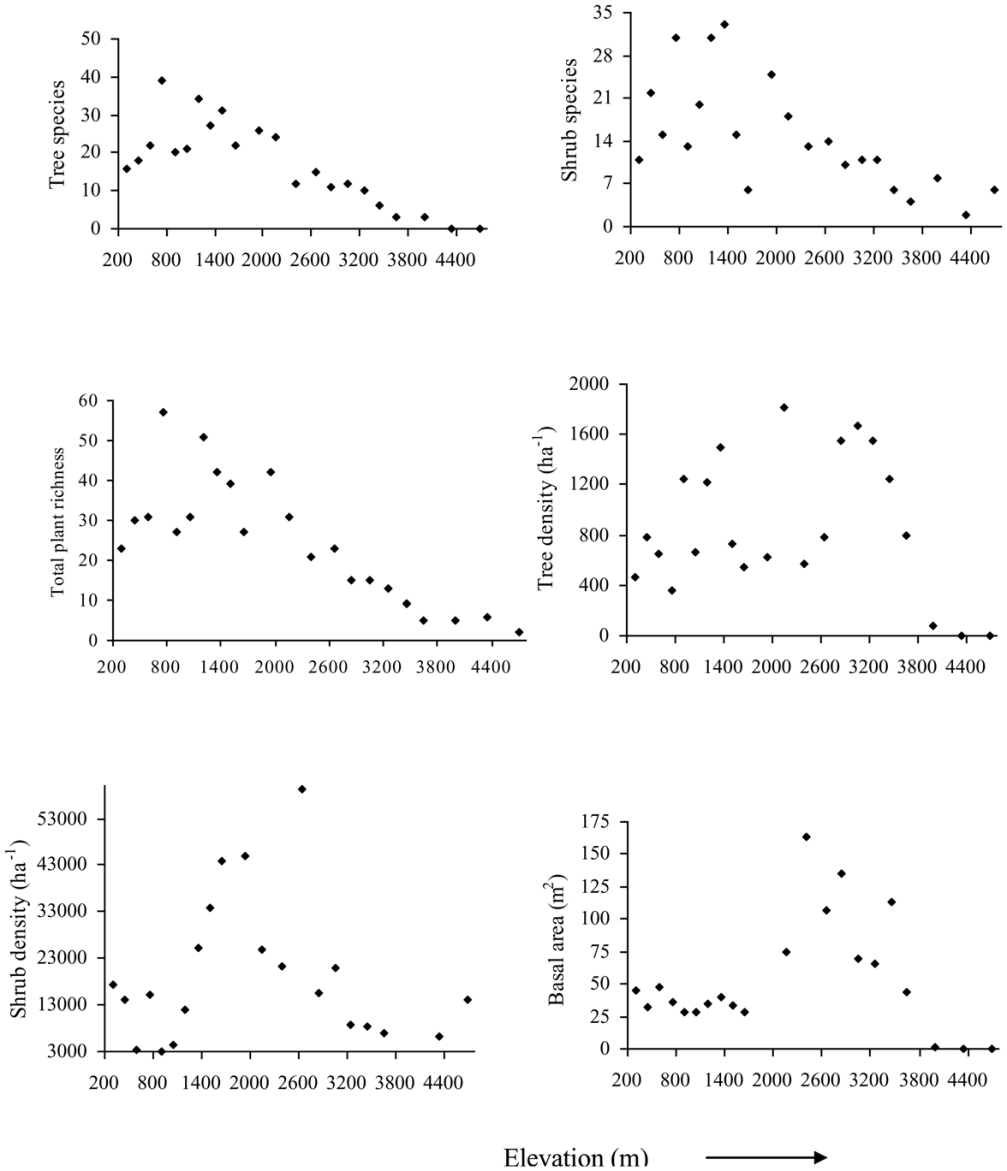
Scatter plot showing the relationship between elevation and vegetation parameters in Sikkim, Eastern Himalaya.

We recorded a total of 216 species of woody plants from the 22 sites. Of the total species observed, 170 were trees and 135 shrubs with 89 species common between trees and shrubs. Species richness of both trees and shrubs followed a hump-shaped relationship with elevation peaking at approximately 1500 m (Tree, r^2^ = 0.71, p<0.05; Shrubs, r^2^ = 0.44, p<0.05; Figure 4). Combined richness of trees and shrubs peaked at approximately1000 m. Shrub density also followed unimodal pattern with a peak at 1500 m, but tree density did not vary systematically with elevation. Basal area of trees was greatest at 1900 m elevation (Figure 4).

### Is there evidence of a mid-domain effect?

**Figure 5:**
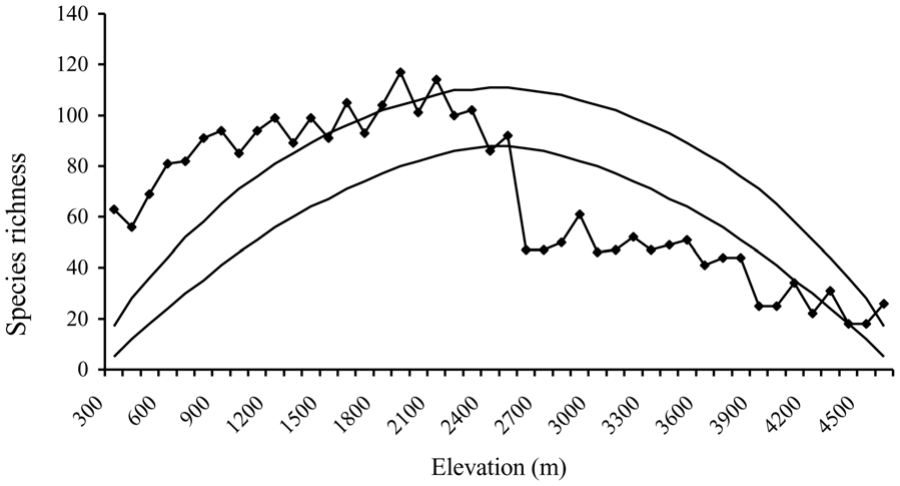
Mid-domain effect null model. Bird species richness curve (line with squares) along the elevation gradient of Sikkim, Eastern Himalaya with 95% simulation curves (lines without markers) obtained using empirical range sizes through the programme mid- domain null [30].

We found, at best, limited support for a mid-domain effect. The curves were asymmetrical, and thus differed from mid-domain predictions (Figure 5). A comparison of the empirical data with the 95% prediction curves obtained from 50,000 simulations using range sizes showed that 80% (35/44) of the empirical points occurred outside the predicted range of the null model (Figure 5). Empirical species richness was correlated with the mean of the predicted richness, but only weakly (r^2^ = 0.18; p = 0.003). Additionally, bird species richness did not correlate with the MDE predicted richness (Table 2) and MDE predicted richness (when used as predictor variable for observed bird species richness) fell out of the stepwise regression model (Table 3).

**Table 2:** The r^2^ values and associated *P*-values for simple linear regression between observed species richness, Chao2 estimated richness, rarefied richness and density of birds as a measure of six environmental factors.

Parameters		Observedbird species	Chao2	Rarefied richness	Bird density
MDE richness	r^2^	0.14	0.11	0.14	0.30
	P	0.07	0.13	0.09	0.08
AET	r^2^	**0.25**	**0.29**	**0.35**	**0.06**
	P	0.01	0.009	0.004	0.28
Plant species	r^2^	**0.47**	**0.45**	**0.52**	**0.30**
	P	0.00	0.001	0.00	0.008
Tree density	r^2^	0.02	0.01	0.02	0.02
	P	0.54	0.64	0.53	0.54
Shrub density	r^2^	**0.42**	**0.38**	**0.37**	**0.44**
	P	0.001	0.002	0.003	0.001
BA	r^2^	**0.18**	**0.11**	**0.18**	**0.32**
	P	0.05	0.13	0.05	0.006

MDE - Mid-domain effect; AET - Actual evapotranspiration; BA - Basal area of trees. Significant (*P*≤0.05) r^2^ values are shown in bold font.

**Table 3:** Result of stepwise multiple regressions with bird species richness and bird density as response variable and MDE predicted richness, AET, plant species richness, tree density, shrub density and BA of trees as predictor variable.

Model: Bird species ∼ AET + Shrub density					
<none>		Df	Sum of Sq	RSS	AIC
		-	-	2569.3	110.73
AET		1	2037.4	4606.7	121.57
Shrub Density		1	3336.3	5905.6	127.04
Residuals	Min	1Q	Median	3Q	Max
	-7.078	-5.486	-1.185	1.414	32.487
Coefficients		Estimate	Std. Error	t value	Pr (>|t|)
(Intercept)		1.843	6.395	2.881	0.009**
AET		0.0198	0.0051	3.882	0.001**
Shrub Density		0.000828	0.000166	4.967	0.00008***

Model: Bird density ∼ Plant richness + Shrub density + BA					
<none>		Df	Sum of Sq	RSS	AIC
		-	-	1796.4	104.86
Plant		1	762.06	2558.4	110.63
Shrub Density		1	597.03	2393.4	109.17
BA		1	505.85	2302.2	108.31
Residuals	Min	1Q	Median	3Q	Max
	-13.8160	-6.2631	0.1712	3.6711	22.8234
Coefficients		Estimate	Std. Error	t value	Pr (>|t|)
(Intercept)		0.0883	4.669	0.019	0.9851
Plant		0.4119	0.149	2.763	0.0128 *
Shrub Density		0.0004	0.0001	2.446	0.0250 *
BA		0.0049	0.0021	2.251	0.0371 *

MDE - Mid-domain effect; AET- Actual evapotranspiration; BA- Basal area of trees.

Only final model is presented here. Significant codes: ≤0 ‘***’ ≤0.001 ‘**’ ≤0.01 ‘*’.

What factors are correlated with richness?

The r^2^ values and associated p-values for simple linear regression between bird species richness (observed and the Chao2 estimate), rarefied richness and density as a function of six environmental factors are shown in Table 2. AET, plant species richness, and shrub density were all positively correlated with bird species richness (observed, estimated and rarefied), whereas bird density was correlated with plant species richness, shrub density and basal area. In the stepwise regression model, AET and shrub density remained as the most important factors for bird species richness along the elevational gradient (Table 3). Plant species richness, shrub density and basal area were most strongly correlated with bird density.

**Figure 6:**
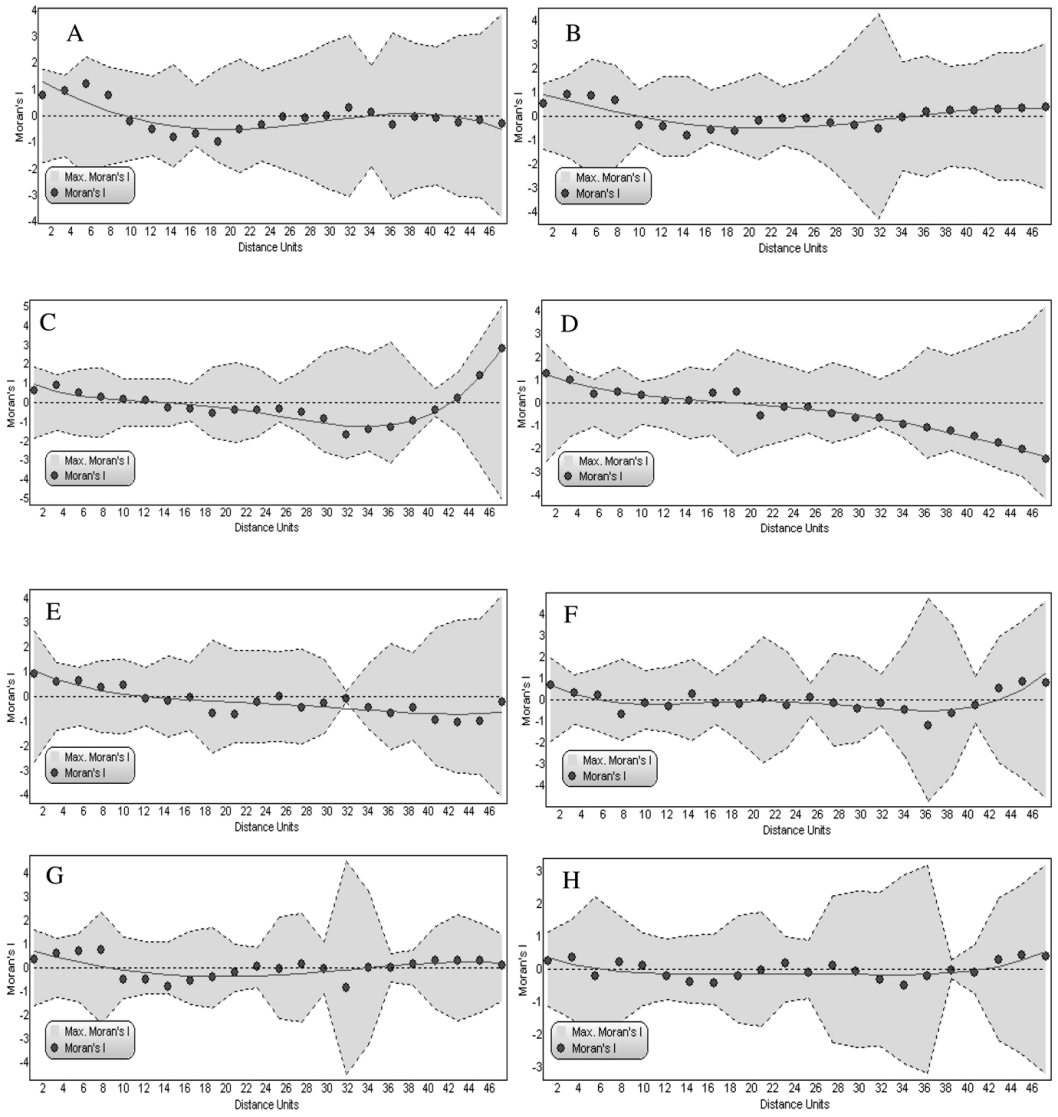
Spatial correlograms for birds, AET and habitat variables along the elevation gradient of Sikkim, Eastern Himalaya. Correlograms represents (A) observed bird species richness, (B) bird density, (C) mid-domain effect predicted richness, (D) actual evapotranspiration, (E) plant species richness, (F) tree density, (G) shrub density and (H) basal area of trees.

The spatial correlogram for species richness (Figure 6) indicated that richness was positively spatially autocorrelated up to 10 distance classes. Moran's I decreased beyond that point with negative or no correlation but the values were not statistically significant. Bird density also followed similar trend. For the suite of environmental variables, positive spatial autocorrelation appeared up to a few distance classes in all the cases with decline in Moran's I index towards higher distance classes (Figure 6). For MDE predicted richness, tree density, and tree basal area, positive autocorrelation reappeared in the largest distance classes but for AET and plant species richness the Moran's I index declined towards larger distance classes with a negative autocorrelation coefficient.

### Are range size and elevation correlated?

**Figure 7:**
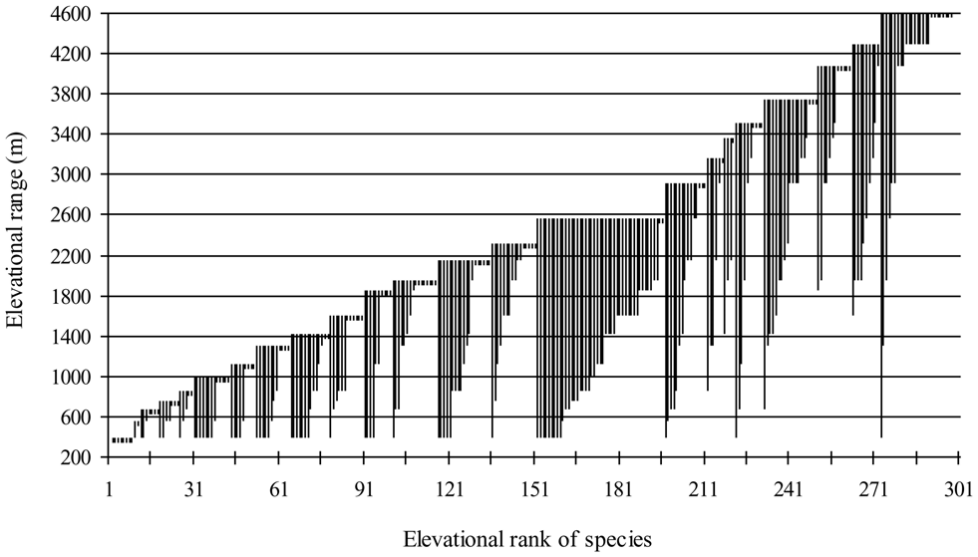
Elevational range profiles of birds of Sikkim, Eastern Himalaya. Vertical bars indicate maximum and minimum elevational limits of each species.

**Figure 8:**
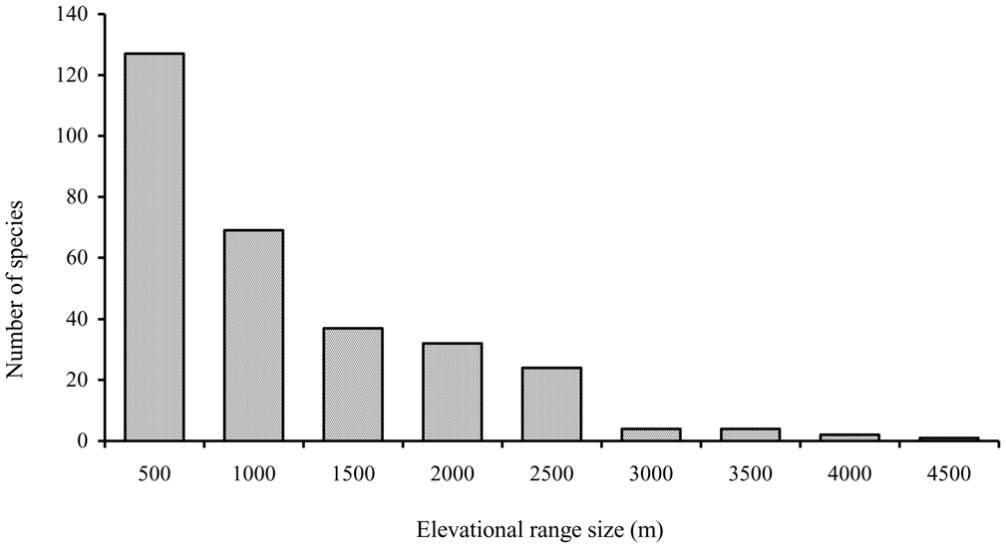
Elevational range-size distribution of birds of Sikkim, Eastern Himalaya.

Elevational range profiles of the birds of Eastern Himalaya showed that most species occupied very narrow elevational ranges along the gradient (Figure 7). Ninety bird species were restricted within 1800 m elevation, whereas 200 species occurred below 2600 m, and 40 species occurred only above 3000 m (Figure 7). Approximately 42% (125) of the bird species had elevational ranges of <500 m, and 30% (90 species) were detected at only a single elevation. Thirty five species had range sizes of more than 2000 m (Figure 8). Only one species (White-capped Water Redstart Chaimarrornis leucocephalus) occurred at each site in the gradient (elevational range = 4500 m). The range sizes of low elevation species (especially those occurring below 1800 m elevation) tended to decrease with elevation (r = -0.34, p < 0.01), whereas range sizes of high elevation species tended to increase with elevation (r = 0.37, p < 0.01).

## Discussion

Along one of the most extensive elevational gradients in the world, we found that the species richness of birds in Eastern Himalaya displayed a distinct mid-elevation peak in species richness and density. Such a pattern is frequently documented in birds (see [6]), small mammals [8], [23], [30], [52], herpetofauna [53], [54], invertebrates [55], [56] and plants [18], [57], [58], [59]. Other taxa in the Himalayas and nearby regions also exhibit mid-elevation peaks in species richness: plant diversity in the Central Himalaya, Nepal and Western Himalaya, India [42], [58], [60] and small mammal diversity in the Mt. Qilian, China [61]. Clearly, the pattern is common, but what causes it?

One idea is that perhaps the geometry of bounded domains or available area accounts for the mid-elevation peak in richness found here [27], [29], [62]. However, we found little support for the MDE: only about 20% of the observed values of species richness occurred within the 95% prediction curve of the null model (Figure 5) and MDE predicted richness was not correlated with avian richness (Table 2). In some cases, the MDE can account for most of the variation in species richness [18], [29], [63]. But in others it accounts for very little, or no variation in species richness (e.g., [6], [62], [64], [65]). Determining the circumstances for when the MDE does, and does not, account for variation in species richness is an important challenge for biogeographers and macroecologists [62]. We also think that available area is likely not important here. Furthermore, the most recent synthetic analysis at global scales found no support for the idea that area influences avian species richness along elevational gradients (see [6]).

Various processes such as climate, productivity, habitat heterogeneity and mass effects have been proposed to explain elevational distributions of species [1], [9], [22], [66]. Though we did not test the entire suite of possible factors that could shape the pattern of bird diversity in the Eastern Himalaya, we found strong support for climatic and habitat variables. In particular, when we examined the potential correlates of species richness in isolation of one another using simple linear regressions, we found that both species richness and density of birds were positively and strongly correlated with AET, plant species richness and shrub density. Habitat heterogeneity and productivity are often correlated with bird species richness at various geographical scales [67]–[71]. The diverse habitat with complex vegetation structure at mid-elevations in the Eastern Himalaya has relatively higher productivity, which would have caused peaks in species richness and abundance of birds.

Many studies have examined how productivity might influence diversity [2], [22], and even in eastern Asia, there appears to be a relationship between primary productivity and bird species richness [72], [73]. However, some contention persists about both the shape of the relationship between diversity and productivity [22] and how exactly more productivity might lead to higher species richness [74]. The most frequently posited mechanism linking productivity to diversity is something like the ‘More Individuals Hypothesis’ [75] or ‘species-energy’ theory. The basic idea is that more productivity leads to more individuals, and with more individuals, species richness is also higher, either because of reduced extinction probabilities or simply because of the sampling effect. However, it is unclear exactly why more energy should lead to more individuals of different species rather than simply more individuals of the same species. Indeed, in our study, when we removed the effect of ‘more individuals’ by rarefaction, there was still a strong and positive correlation between rarefied richness and AET.

Evolutionary and historical events such as geographic isolation, dispersal, speciation and endemism could shape elevational diversity in montane regions [24], [71], [76]–[80]. It is hypothesized that several speciation events and subsequent dispersal into Himalayas occurred due to the formation of new habitats by climatic changes in the past [79], [81]. Endemism appears to be lower in the Himalayas relative to other montane regions [82] and indeed in all other global hotspots [83]. Furthermore, recent work argues that speciation alone is not likely to drive the pattern we describe here because speciation is low within the Himalaya due to an apparent lack of potential barriers [79]. Since Eastern Himalaya is located at the transition belt of Oriental and Palaearctic zoogeographical realms and Indian, Indochinese and Indomalayan regions [84], the avifauna in the study region could consist of immigrants from these realms and regions due to dispersal of species. While it would be unwarranted at this stage to discard endemism and speciation due to a dearth of empirical studies, further work could address these issues. In particular, applying phylogenetic analyses for the birds in the Eastern Himalayan region would clearly allow for a better evaluation of how (or whether) historical and evolutionary factors influence species richness (e.g., [26], [80], [85]). However, the lack of robust phylogenetic hypotheses for many of these taxa examined here prevented us from pursuing this line of research.

Variation in species richness and density are rarely, if ever, wholly explained by a single factor [58]. And species richness varies in peculiar ways among taxa, even on the same elevational gradient [11]. In our case, we found that climatic and habitat factors accounted for most of the variation in the density and species richness of birds. It is of course not surprising that multiple factors can shape diversity gradients, and perhaps that is to be expected. What is somewhat surprising, however, is that these same factors accounted for variation in every attribute of the avian communities examined here – observed richness, the Chao2 estimate of diversity, rarefied richness and density.

The decline in both species richness and density of birds above 2500 m is striking, and suggests an abrupt change in some factor or suite of factors that limits birds. The stature of the forest decreases dramatically at about this point, and the climatic conditions become increasingly severe beyond 2500 m in the region; both of these changes could cause declines in abundance and size distribution of invertebrates and scarcity of other food items for birds [15]. One potential criticism of our study is that we did not continue to sample avian communities above 4700 m elevation. However, we note that the decline in avian richness from 3000 m to 4700 m is very low (32 species at 3050 m and 27 species at 4700 m). While there are essentially a very few bird species above this elevation in this part of the Himalayas and, even if a few transient species were detected, their presence would not have qualitatively changed the overall patterns we document here.

Rapoport's rule (extended to elevational gradients by Stevens [33]) suggests that range size of species should increase with increasing elevation. Although range size of high elevation species in our case tended to increase with elevation, the relation was weak, and the ranges of low-elevation species actually decreased with increasing elevation. Hence, we find little evidence to support Rapoport's rule for the birds of Sikkim region of the Himalaya, indicating that Rapoport's rule does not explain the elevational pattern of birds in the eastern Himalaya. Rapoport's rule has invited criticisms and whether this rule is a general phenomena is an open question in biogeography [86], [87].

Most bird species in this study exhibited very narrow elevational ranges. Interestingly, of 297 species, only one species (White-capped Water Redstart Chaimarrornis leucocephalus) occurred at all 22 sites. Most occurred at only a few sites, suggesting that range sizes are extremely limited in this system, probably by a combination of dispersal ability, particular habitat associations, competition, or environmental tolerance [88], [89], [90]. Most species here appear to be habitat specialists, either restricted to a handful of sites or a single vegetation zone. Those species with larger elevational ranges tended to be omnivores. For example, the omnivorous birds Blue Whistling Thrush (Myophonus caeruleus) and White-capped Water Redstart occupied extensive ranges along the gradient, whereas a true frugivore Pin-tailed Green Pigeon (Treron apicauda) was present at only a single elevation site. Playback experiments, coupled with physiological tolerance and behavioral observations about the degree of specialization among species would help elucidate the factors that limit the ranges of species along this extreme elevational gradient [91]. However, in the absence of such exhaustive studies, incorporating phylogenetic analyses as a short cut to understanding the interplay between interspecific interactions and climatic tolerance (e.g., [85]) would clearly be an important next step.

In sum, along one of the longest elevational gradients in the world, we found that bird species richness and density showed hump-shaped relationship with elevation, peaking at approximately 2000 m, in the Eastern Himalayan region. The variation in richness and density was correlated strongly with both productivity and habitat rather than geometric constraints. The small elevational ranges of species along the gradient suggest that conservation efforts should consider the entire gradient rather than just portions of it.

## Supporting Information

Figure S1 **Species accumulation curves of birds.** Curves based on number of point counts in different elevational transects in Sikkim, Eastern Himalaya. Numbers in the figures indicate elevation (m) of the sampling site.
(TIF)

Figure S2 **Elevational variation of rarefied bird species richness.** Species richness observed when rarefied to 72 point counts along elevational transects in Sikkim, Eastern Himalaya.
(TIF)

Figure S3 **Elevational variation of rarefied bird species richness.** Species richness observed when rarefied to 15 individuals from six point counts from each site along elevational transects in Sikkim, Eastern Himalaya.
(TIF)
