# Supplementary figures and images for: Elevational Gradients in Bird Diversity in the Eastern Himalaya: An Evaluation of Distribution Patterns and Their Underlying Mechanisms
Source: PLoS One. 2011 Dec 13;6(12):e29097. doi: 10.1371/journal.pone.0029097 (PMC3236786; doi:10.1371/journal.pone.0029097)

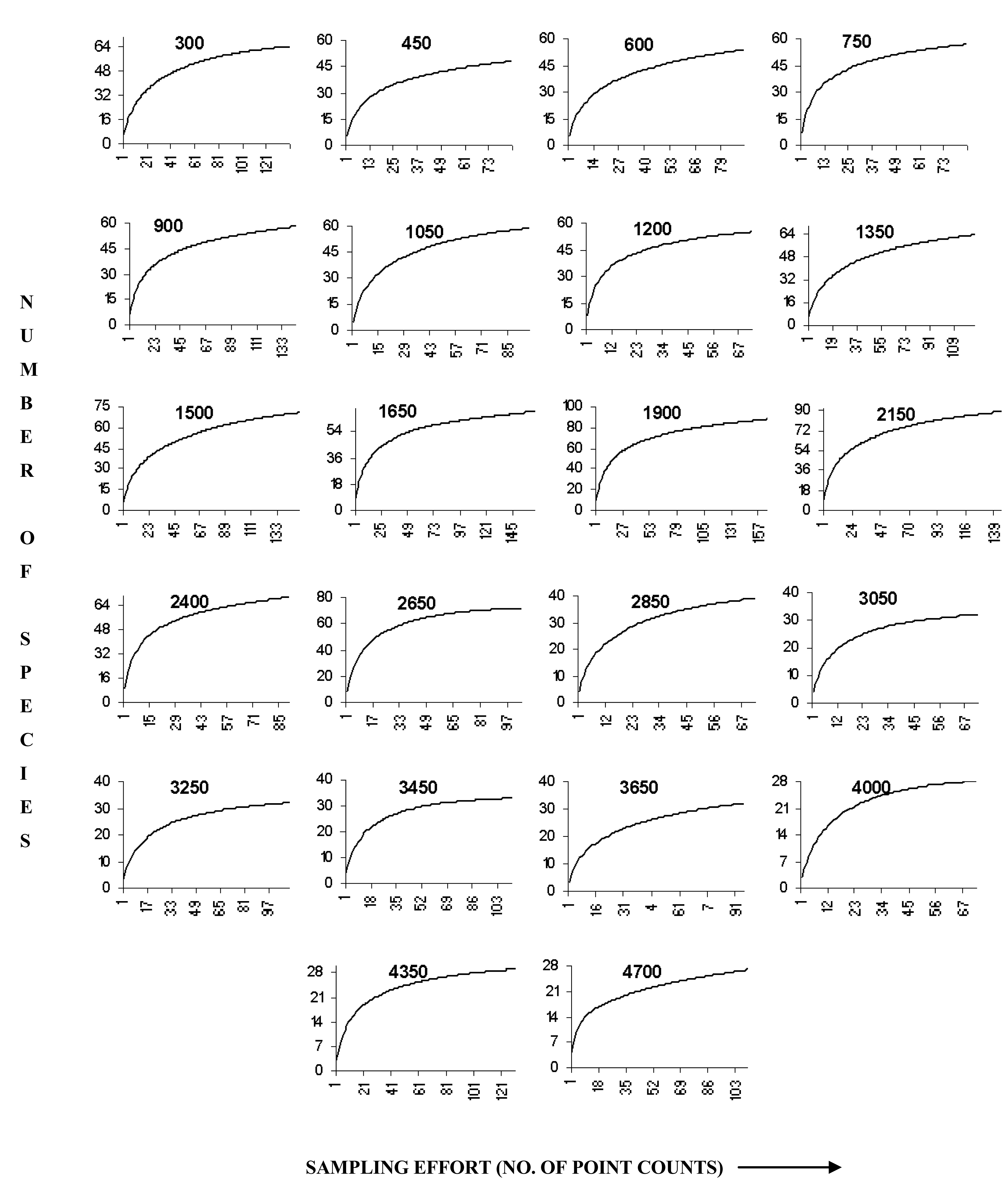

Supplement: Figure S1 — Species accumulation curves of birds. Curves based on number of point counts in different elevational transects in Sikkim, Eastern Himalaya. Numbers in the figures indicate elevation (m) of the sampling site. (TIF) [file pone.0029097.s001.tif]

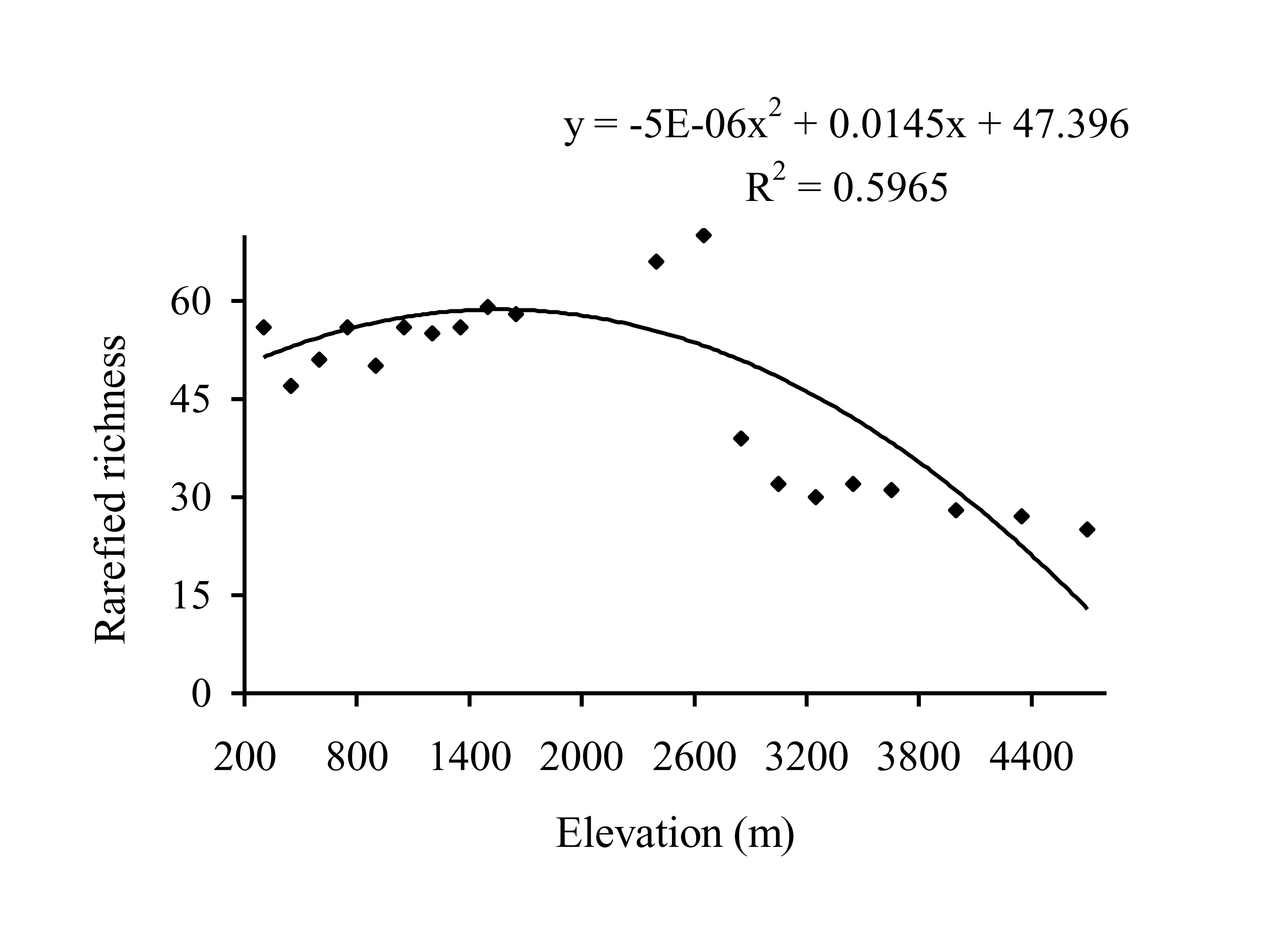

Supplement: Figure S2 — Elevational variation of rarefied bird species richness. Species richness observed when rarefied to 72 point counts along elevational transects in Sikkim, Eastern Himalaya. (TIF) [file pone.0029097.s002.tif]

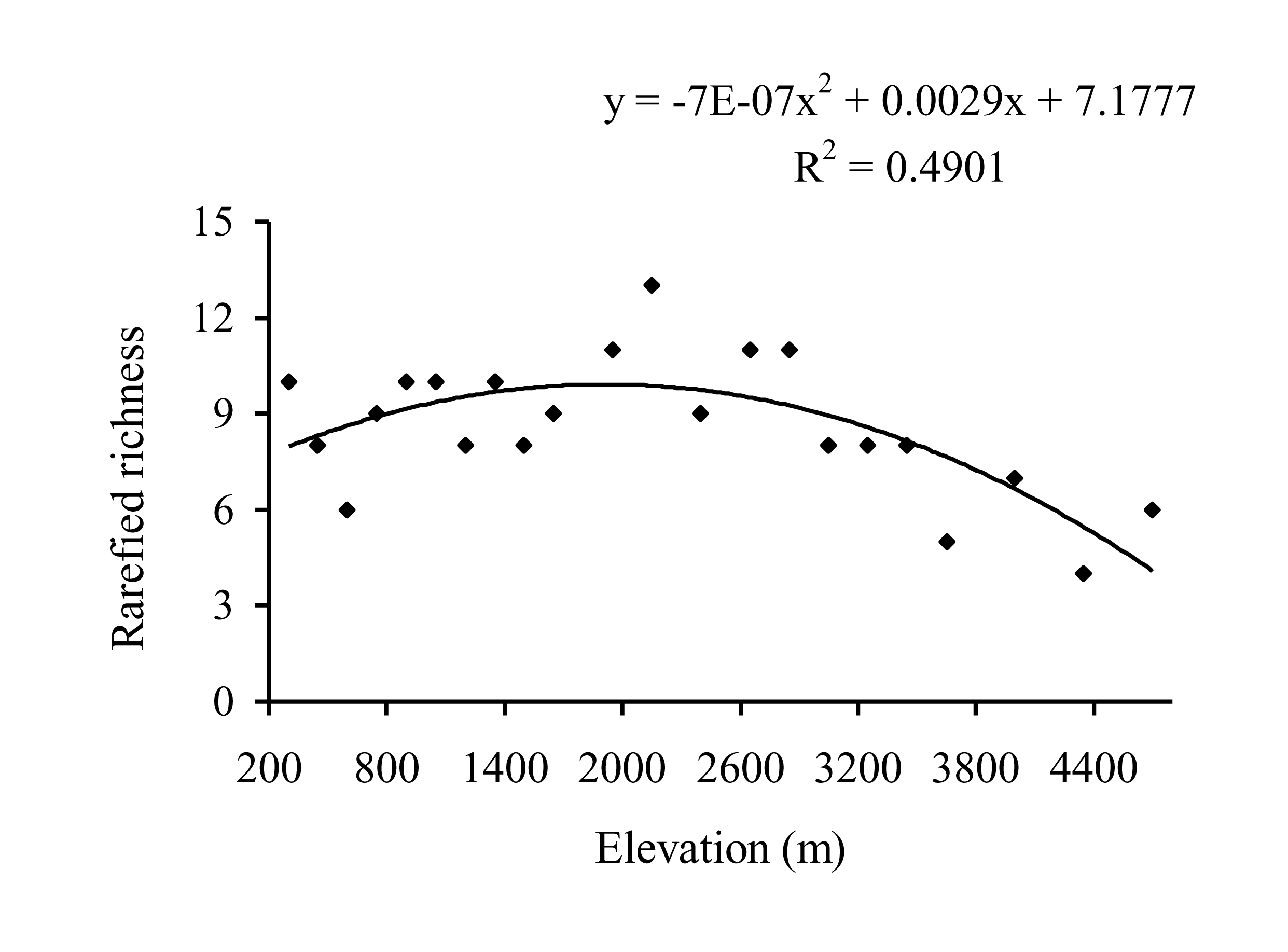

Supplement: Figure S3 — Elevational variation of rarefied bird species richness. Species richness observed when rarefied to 15 individuals from six point counts from each site along elevational transects in Sikkim, Eastern Himalaya. (TIF) [file pone.0029097.s003.tif]
